# SNP-VISTA: An interactive SNP visualization tool

**DOI:** 10.1186/1471-2105-6-292

**Published:** 2005-12-08

**Authors:** Nameeta Shah, Michael V Teplitsky, Simon Minovitsky, Len A Pennacchio, Philip Hugenholtz, Bernd Hamann, Inna L Dubchak

**Affiliations:** 1 Institute for Data Analysis and Visualization, (IDAV), Department of Computer Science, University of California, Davis, One Shields Ave., Davis, CA 95616, USA; 2 Genomics Division, Lawrence Berkeley National Laboratory, One Cyclotron Road, Berkeley, CA, 94720, USA; 3 DOE Joint Genome Institute, 2800 Mitchell Drive, Walnut Creek, CA 94598, USA

## Abstract

**Background:**

Recent advances in sequencing technologies promise to provide a better understanding of the genetics of human disease as well as the evolution of microbial populations. Single Nucleotide Polymorphisms (SNPs) are established genetic markers that aid in the identification of loci affecting quantitative traits and/or disease in a wide variety of eukaryotic species. With today's technological capabilities, it has become possible to re-sequence a large set of appropriate candidate genes in individuals with a given disease in an attempt to identify causative mutations. In addition, SNPs have been used extensively in efforts to study the evolution of microbial populations, and the recent application of random shotgun sequencing to environmental samples enables more extensive SNP analysis of co-occurring and co-evolving microbial populations. The program is available at [1].

**Results:**

We have developed and present two modifications of an interactive visualization tool, SNP-VISTA, to aid in the analyses of the following types of data: A. Large-scale re-sequence data of disease-related genes for discovery of associated and/or causative alleles (GeneSNP-VISTA). B. Massive amounts of ecogenomics data for studying homologous recombination in microbial populations (EcoSNP-VISTA). The main features and capabilities of SNP-VISTA are: 1) mapping of SNPs to gene structure; 2) classification of SNPs, based on their location in the gene, frequency of occurrence in samples and allele composition; 3) clustering, based on user-defined subsets of SNPs, highlighting haplotypes as well as recombinant sequences; 4) integration of protein evolutionary conservation visualization; and 5) display of automatically calculated recombination points that are user-editable.

**Conclusion:**

The main strength of SNP-VISTA is its graphical interface and use of visual representations, which support interactive exploration and hence better understanding of large-scale SNP data by the user.

## Background

Polymorphisms are nucleotide differences in genomic DNA sequences that naturally occur within a population. A single nucleotide substitution is called a single nucleotide polymorphism (SNP) and these variants occur at a frequency of approximately one every 1,000 bases in humans [2]. SNPs are established genetic markers that aid in the identification of loci affecting quantitative traits and/or disease in a wide variety of eukaryote species. The recent completion of a single version of the human genome [3,4] has now provided the substrates for direct comparison of individuals in both health and disease. Ideally, to better understand the genetic contributions to severe diseases, one would obtain the entire human genome sequence for all disease-carrying individuals for comparison to unaffected control groups. While these complete datasets are not readily obtainable today, a strategy that is currently approachable is the re-sequencing of a large set of appropriate candidate genes in individuals with a given disease to screen for potential causative/susceptibility alleles. However, one ongoing challenge remains in the visualization of such datasets by end users to thereby derive biological insights.

In addition, SNPs have been used extensively in efforts to study the evolution of microbial populations. Such efforts have largely been confined to multi-locus sequence typing of clinical isolates of species such as *Neisseria meningitidis *and S.*taphylococcus aureus *[5]. However, the recent application of random shotgun sequencing to environmental samples [6-8] enable more extensive SNP analysis of co-occurring and co-evolving microbial populations. An intriguing finding in one study [6] was the mosaic nature of the genomes of an archaeal population inferred to be the result of extensive homologous recombination of three ancestral strains. This observation was based on the manual analysis of a small subset of the data (ca. 40,000 basepairs) and remains to be verified across the whole genome. Tools to analyze these type of data are also in their infancy.

Manipulation, cross-referencing, and haplotype viewing of SNP data are essential for quality assessment and identification of variants associated with genetic disease [9]. The display and interpretation of large genotype data sets can be simplified by using a graphical display. Several software tools have been developed to assist researchers to carry out this task. A visual genotype (VG2) display [10,11] proved to be useful in showing raw datasets of individuals' genotype data. This format presents all data in an array of samples (rows) × polymorphic sites (columns) and encodes each diallelic polymorphism according to a general color scheme. This array format allows one to visually inspect the data across both individual's diplotypes and polymorphic sites to make comparisons and identify correlated sub-datasets. Another program, ViewGene [12], was developed as a flexible tool that utilizes input data and constructs an assembly reference scaffold that can be viewed through a simple graphical interface. Polymorphisms generated from many sources can be added to this scaffold with a variety of options to control what is displayed. Large amounts of polymorphism data can be organized so that patterns and haplotypes can be readily discerned. Two other tools, Genewindow [13] and SNPper [14], were recently developed as Web-based applications to mark variations on the human DNA. A final software system for automated and visual analysis of functionally annotated haplotypes, HapScope [15], displays genomic structure with haplotype information in an integrated environment, providing alternative views for assessing genetic and functional correlation.

Although these tools provide a number of valuable options for the scientist, some user requirements are not satisfied. VG2 uses simple but effective representations to show genotype data with SNP classification and organizes the data using hierarchical clustering. The major drawbacks of this tool are its static display, lack of provision for details on demand and lack of capabilities to map SNPs to genomic structure. ViewGene provides a simple interface for analyzing sequence data to locate regions favorable to re-sequencing but is limited in its capabilities for post-processing of SNP data. HapScope consists of valuable haplotype analysis methods along with interactive visualization, but its major focus is the presentation of results from haplotype analysis. Our goal was to develop exploration tools for discovery of disease-related mutations from re-sequencing data.

It is important to note that most experiments in SNP research are exploratory in nature, and it has become essential to provide the scientific community with an advanced SNP exploration tools. With SNP data growing as a result of large-scale gene re-sequencing and ecogenomics projects, there exists a need to overcome limitations of current SNP analysis tools. We present an interactive visualization tool, which aids scientists in generating hypotheses from large-scale SNP data.

## Implementation

SNP-VISTA is implemented as a stand-alone Java application using JBuilder [16] as a development environment. SNP-VISTA uses clustering software, Levenshtein [17], which is bundled with the package. Automatic recombination points are calculated using a C++ program that can be invoked from the Java application.

## Results

SNP-VISTA is available in two versions, as GeneSNP-VISTA or EcoSNP-VISTA, each tailored for a specific application. We describe each version in the next two sections.

### GeneSNP-VISTA: Visualization of disease-related mutations in genes

We use the ABO blood group gene (transferase A, alpha 1-3-N-acetylgalactosaminyltransferase; transferase B, alpha 1.3.galactosyltransferase) from the Finished Genes Page of SeattleSNPs [18] to demonstrate our tool's capabilities.

The program requires the following files as input:

#### Reference sequence

This file contains the DNA sequence of the gene in FASTA format [19].

#### Annotation file

A tab-delimited file with annotation for exons and coding sequence (cds) in the following format:

<exon/cds><tab><start><tab><end>

If the coding sequence is not specified explicitly then exons are merged to obtain the coding sequence.

#### SNP data

A tab-delimited file with four fields on each line, in the format:

<Site Position><tab><Sample ID><tab><Allele 1><tab><Allele 2>

#### Protein alignment

This file contains the protein alignment in multi-FASTA format. The first protein in the file must be the protein corresponding to the gene given in the reference sequence.

Sample input files are available on the website [20].

GeneSNP-VISTA supports the following applications:

#### Mapping of SNPs to gene structure

**Figure 1:**
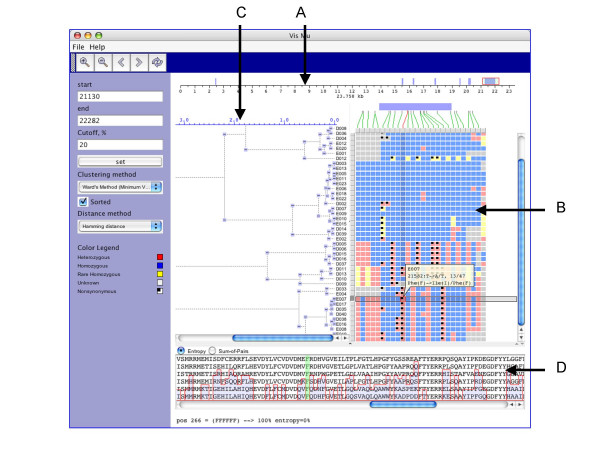
GeneSNP-VISTA screenshot for the ABO blood group gene (transferase A, alpha 1-3-N-acetylgalactosaminyltransferase; transferase B, alpha 1.3.galactosyltransferase). A. Coordinate bar showing gene structure. The ABO gene consists of 23,758 basepairs. Seven exons are displayed as blue rectangles. The red rectangle is a user-selected region. B. SNPs are represented as an array of samples (rows) × polymorphic sites (columns), where each cell is colored based on the SNP classification. Blue is used for individuals homozygous for the common SNP allele, yellow color is used for individuals homozygous for the rare SNP allele, red is for individuals heterozygous for the SNP, and a black dot is used for non-synonymous SNP. C. Clustering results are shown as a hierarchical tree, where each node can be collapsed or expanded. D. Window displaying protein alignment. The display is linked with the non-synonymous SNP selected by the user.

A SNP can be located in a UTR, exon, intron or splice site. Such information about the location of SNPs is valuable to biologists. To illustrate this feature, we mapped SNPs with the ABO dataset to its gene structure as shown in Figure 1A. A coordinate bar represents the *ABO *blood group gene, which is 23,758 base pairs long and has seven exons that are shown by blue rectangles. The red rectangle is the user-selected sub-region of the gene. Green lines show the exact location of each SNP in the gene. On mouse over the connecting line is highlighted in red.

#### Classification of SNPs

A SNP can be found in several genotypic forms in a given individual (homozygous or heterozygous for either of the two nucleotide states). In addition, SNPs in coding sequence can come in two forms; synonymous (those that do not change an amino acid) and non-synonymous (those that do change an amino acid)]. Our program bins SNPs into these various categories and uses different colors for each class of SNPs The graphical representation is similar to VG2, where selected data is represented as an array of samples (rows) × polymorphic sites (columns), and each cell is colored depending on the classification of a given SNP based on their location in the gene, frequency of occurrence in samples and allele composition (Figure 1B). Upon mouse over, detailed information (sample id, position, frequency, etc.) about the selected SNP is displayed in a semi-transparent callout.

#### Clustering

Clustering of samples based on the patterns of SNPs allows a user to navigate through the data. We use Levenshtein software package to perform hierarchical clustering. Clustering can be performed using all the SNPs in the data or a user-selected subset. GeneSNP-VISTA displays the hierarchical tree (Figure 1C) where each node can be collapsed or expanded.

#### Integration of multiple alignments of homologous proteins in different species

One of the approaches to assess how functional a SNP might be when it changes an amino acid is to investigate the conservation of that amino acid across multiple additional species. A SNP causing change in a conserved amino acid would be predicted to be more likely to be a functional change since over evolutionary time this amino acid has resisted genetic drift. Integration of multiple alignments of homologous proteins allows a scientist to determine whether a SNP has caused a conserved amino acid to change. GeneSNP-VISTA displays the protein alignment along with an entropy or sum-of-pairs similarity score in the protein alignment window (Figure 1D). When a user selects a non-synonymous SNP, the corresponding amino acid is highlighted in green. In Figure 1, the user has selected a heterozygous non-synonymous SNP in the last exon, which changes the amino acid Phenylalanine (F) to Isoleucine (I). The protein alignment window shows the conservation of this amino acid, which is 100% conserved. Consistent with our finding, the SIFT program [21] predicts this position as intolerant, and the Polyphen program [22] deems it as probably damaging change (see results at [23].

### EcoSNP-VISTA: Discovery of recombination points in microbial populations

As our test bed, we have used the acid mine drainage [6] dataset that is publicly available at [24].

The following files are needed as input:

#### Alignment data

This file contains the blast output obtained by blasting the consensus sequence against all reads in the database.

#### Annotation file

This file is similar to the GeneSNP-VISTA annotation file, and it has the following format:

<exon/cds><tab><start><tab><end>

#### Recombination points (optional)

A tab-delimited file with four fields on each line, in the format:

<Read name><tab><Position>

Sample input files are available at [25].

The following modifications are made to GeneSNP-VISTA to handle ecogenomics data:

#### Nucleotide-based color scheme

**Figure 2:**
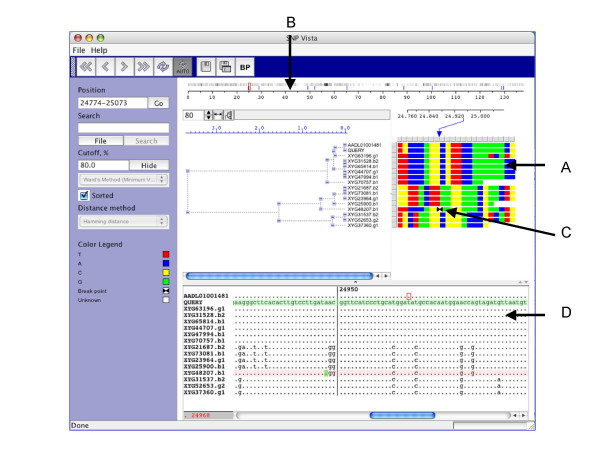
EcoSNP-VISTA screenshot of scaffold 1 of the microbial genome of ferroplasma II [6]. A. SNPs are represented as an array of reads (rows) × polymorphic sites (columns), where each cell is colored based on the nucleotide. Red is used for nucleotide T (Thyamine), blue is used for nucleotide A (Adenine), yellow is used for nucleotide C (Cytosine), and green is used for nucleotide G (Guanine). B. Coordinate bar providing global view of recombination points shown with blue lines and frequency of SNPs, where black indicates higher frequency. C. Array representation showing exact position of the recombination point with two black triangles. D. Window displaying the BLAST alignment for the selected region.

Each cell in the array is colored based on the nucleotide at the SNP position. Once the reads are clustered this representation allows a user to discern various SNP patterns probably corresponding to different strains (Figure 2A).

#### Recombination point calculation and visualization

A user can provide recombination points, obtained from another program or calculate them by EcoSNP-VISTA. The recombination point calculation is based on the Bellerophon program [26]. Our tool displays recombination points on the coordinate bar using blue lines showing the global view and the frequency of the SNPs (Figure 2B). The array representation also shows the exact position of the recombination point with two black triangles (Figure 2C). The reads can be examined closely in a window as shown in Figure 2D. A user can visually verify the recombination points and accept or reject them. It is also possible to add a recombination point. Automatic recombination point calculation results typically in a large number of false positives, whereas manual detection of recombination points is a very time-consuming job. EcoSNP-VISTA combines both approaches to provide a feasible method for detecting recombination points.

## Discussion

A major challenge in examining human SNP data is assessing which variants are more likely to be involved in having damaging effects on the structure and function of a gene/protein. GeneSNP-VISTA is an interactive visual tool for highly efficient analysis of large amounts of SNP data to determine a subset that may be of relevance to explaining human disease. As shown in Figure 1, all the information about a SNP (type, location on genomic structure, frequency of occurrence, type of amino acid change and the positions conservation) allows a scientist to determine whether a SNP is a possible causative mutation. By providing a visually integrated representation of SNPs data with genomic structure and protein conservation, GeneSNP-VISTA facilitates the screening of causative mutations from re-sequencing of a large set of appropriate candidate genes in individuals with a given disease.

Adaptation of existing computational methods and development of new ones for effective SNP analysis of co-occurring and co-evolving microbial populations from ecogenomics data poses new challenges. Manual analysis [6] led to interesting results, but such an analysis is time-intensive and becomes prohibitive for whole genome-scale exploration. Automatic methods are not available yet for such an analysis. As an alternative, EcoSNP-VISTA provides a visual interface for semi-automatic analysis of SNPs data from ecogenomics data. As shown in Figure 2, a compact color-coded representation of SNPs data allows a scientist to manually detect recombination points and visually verify automatically calculated recombination points. EcoSNP-VISTA provides insight into homologous recombination in microbial populations and has the potential to guide in the development of computational methods for such analysis.

## Conclusion

We have developed SNP-VISTA, a publicly available interactive visualization tool that assists scientists in the analysis of re-sequence data of disease-related genes for discovery of associated and/or causative alleles and ecogenomics data for studying homologous recombination in microbial populations.

## Availability and requirements

• **Project name: **SNP-VISTA

• **Project home page: **

• **Operating system(s): **MacOSX, Windows, Linux

• **Programming language: **Java

• **Other requirements: **e.g. Java 1.4 or higher

• **License: **Open Source License

• **Any restrictions to use by non-academics: **None

• **Support group: **vista@lbl.gov

## Authors' contributions

NS developed the algorithms and designed the prototype of the program; MT and SM coded and integrated the complete package; LP and PH provided biological insight and actively participated in discussion of the project and writing the paper; BH guided NS at different stages of the project; ID designed and led the project.

## References

[B1] SNP-VISTA Program. http://genome.lbl.gov/vista/snpvista.

[B2] Sachidanandam R, Weissman D, Schmidt SC, Kakol JM, Stein LD, Marth G, Sherry S, Mullikin JC, Mortimore BJ, Willey DL, Hunt SE, Cole CG, Coggill PC, Rice CM, Ning Z, Rogers J, Bentley DR, Kwok PY, Mardis ER, Yeh RT, Schultz B, Cook L, Davenport R, Dante M, Fulton L, Hillier L, Waterston RH, McPherson JD, Gilman B, Schaffner S, Van Etten WJ, Reich D, Higgins J, Daly MJ, Blumenstiel B, Baldwin J, Stange-Thomann N, Zody MC, Linton L, Lander ES, Altshuler D (2001). A map of human genome sequence variation containing 1.42 million single nucleotide polymorphisms. Nature.

[B3] Venter JC, Adams MD, Myers EW, Li PW, Mural RJ, Sutton GG, Smith HO, Yandell M, Evans CA, Holt RA, Gocayne JD, Amanatides P, Ballew RM, Huson DH, Wortman JR, Zhang Q, Kodira CD, Zheng XH, Chen L, Skupski M, Subramanian G, Thomas PD, Zhang J, Gabor Miklos GL, Nelson C, Broder S, Clark AG, Nadeau J, McKusick VA, Zinder N, Levine AJ, Roberts RJ, Simon M, Slayman C, Hunkapiller M, Bolanos R, Delcher A, Dew I, Fasulo D, Flanigan M, Florea L, Halpern A, Hannenhalli S, Kravitz S, Levy S, Mobarry C, Reinert K, Remington K, Abu-Threideh J, Beasley E, Biddick K, Bonazzi V, Brandon R, Cargill M, Chandramouliswaran I, Charlab R, Chaturvedi K, Deng Z, Di Francesco V, Dunn P, Eilbeck K, Evangelista C, Gabrielian AE, Gan W, Ge W, Gong F, Gu Z, Guan P, Heiman TJ, Higgins ME, Ji RR, Ke Z, Ketchum KA, Lai Z, Lei Y, Li Z, Li J, Liang Y, Lin X, Lu F, Merkulov GV, Milshina N, Moore HM, Naik AK, Narayan VA, Neelam B, Nusskern D, Rusch DB, Salzberg S, Shao W, Shue B, Sun J, Wang Z, Wang A, Wang X, Wang J, Wei M, Wides R, Xiao C, Yan C, Yao A, Ye J, Zhan M, Zhang W, Zhang H, Zhao Q, Zheng L, Zhong F, Zhong W, Zhu S, Zhao S, Gilbert D, Baumhueter S, Spier G, Carter C, Cravchik A, Woodage T, Ali F, An H, Awe A, Baldwin D, Baden H, Barnstead M, Barrow I, Beeson K, Busam D, Carver A, Center A, Cheng ML, Curry L, Danaher S, Davenport L, Desilets R, Dietz S, Dodson K, Doup L, Ferriera S, Garg N, Gluecksmann A, Hart B, Haynes J, Haynes C, Heiner C, Hladun S, Hostin D, Houck J, Howland T, Ibegwam C, Johnson J, Kalush F, Kline L, Koduru S, Love A, Mann F, May D, McCawley S, McIntosh T, McMullen I, Moy M, Moy L, Murphy B, Nelson K, Pfannkoch C, Pratts E, Puri V, Qureshi H, Reardon M, Rodriguez R, Rogers YH, Romblad D, Ruhfel B, Scott R, Sitter C, Smallwood M, Stewart E, Strong R, Suh E, Thomas R, Tint NN, Tse S, Vech C, Wang G, Wetter J, Williams S, Williams M, Windsor S, Winn-Deen E, Wolfe K, Zaveri J, Zaveri K, Abril JF, Guigo R, Campbell MJ, Sjolander KV, Karlak B, Kejariwal A, Mi H, Lazareva B, Hatton T, Narechania A, Diemer K, Muruganujan A, Guo N, Sato S, Bafna V, Istrail S, Lippert R, Schwartz R, Walenz B, Yooseph S, Allen D, Basu A, Baxendale J, Blick L, Caminha M, Carnes-Stine J, Caulk P, Chiang YH, Coyne M, Dahlke C, Mays A, Dombroski M, Donnelly M, Ely D, Esparham S, Fosler C, Gire H, Glanowski S, Glasser K, Glodek A, Gorokhov M, Graham K, Gropman B, Harris M, Heil J, Henderson S, Hoover J, Jennings D, Jordan C, Jordan J, Kasha J, Kagan L, Kraft C, Levitsky A, Lewis M, Liu X, Lopez J, Ma D, Majoros W, McDaniel J, Murphy S, Newman M, Nguyen T, Nguyen N, Nodell M, Pan S, Peck J, Peterson M, Rowe W, Sanders R, Scott J, Simpson M, Smith T, Sprague A, Stockwell T, Turner R, Venter E, Wang M, Wen M, Wu D, Wu M, Xia A, Zandieh A, Zhu X (2001). The sequence of the human genome. Science.

[B4] The Human Genome Consortium (2001). Initial sequencing and analysis of the human genome. Nature.

[B5] Spratt BG, Zhang Q, Jones DM, Hutchison A, Brannigan JA, Dowson CG (1989). Recruitment of a Penicillin-Binding Protein Gene from Neisseria flavescens during the Emergence of Penicillin Resistance in Neisseria meningitidis. PNAS.

[B6] Tyson GW, Chapman J, Hugenholtz P, Allen EE, Ram RJ, Richardson PM, Solovyev VV, Rubin EM, Rokhsar DS, Banfield JF (2004). Community structure and metabolism through reconstruction of microbial genomes from the environment. Nature.

[B7] Venter JC, Remington K, Heidelberg JF, Halpern AL, Rusch D, Eisen JA, Wu D, Paulsen I, Nelson KE, Nelson W, Fouts DE, Levy S, Knap AH, Lomas MW, Nealson K, White O, Peterson J, Hoffman J, Parsons R, Baden-Tillson H, Pfannkoch C, Rogers YH, Smith HO (2004). Environmental genome shotgun sequencing of the Sargasso Sea. Science.

[B8] Tringe SG, von Mering C, Kobayashi A, Salamov AA, Chen K, Chang HW, Podar M, Short JM, Mathur EJ, Detter JC, Bork P, Hugenholtz P, Rubin EM (2005). Comparative metagenomics of microbial communities. Science.

[B9] The International HapMap Consortium (2003). The International HapMap Project. Nature.

[B10] Nickerson DA, Taylor SL, Weiss KM, Clark AG, Hutchinson RG, Stengard J, Salomaa V, Vartiainen E, Boerwinkle E, Sing CF (1998). DNA sequence diversity in a 9.7-kb region of the human lipoprotein lipase gene. Nature Genetics.

[B11] Reider MJ, Taylor SL, Clark AG, Nickerson DA (1999). Sequence variation in the human angiotensin converting enzyme. Nature Genetics.

[B12] Kashuk C, SenGupta S, Eichler E, Chakravarti A (2002). ViewGene: A graphical tool for polymorphism visualization and characterization. Genome Research.

[B13] Riva A, Kohane IS (2002). SNPper: retrieval and analysis of human SNPs. Bioinformatics.

[B14] Staats B, Qi L, Beerman M, Sicotte H, Burdett LA, Packer B, Chanock SJ, Yeager M (2005). Genewindow: an interactive tool for visualization of genomic variation. Nature Genetics.

[B15] Zhang J, Rowe WL, Struewing JP, Buetow KH (2002). HapScope: A software system for automated and visual analysis of functionally annotated haplotypes. Nucleic Acids Research.

[B16] Borland : JBuilder Web site. http://www.borland.com/us/products/jbuilder/index.html.

[B17] RuG/L04 – dialectometrics & cartography Web site. http://odur.let.rug.nl/~kleiweg/levenshtein/index.html.

[B18] SeattleSNPs Web site. http://pga.mbt.washington.edu/.

[B19] EBI Help Web site. http://www.ebi.ac.uk/help/formats_frame.html.

[B20] GeneSNP-VISTA program. http://genome.lbl.gov/vista/GeneSNP-VISTA/.

[B21] Ng PC, Henikoff S (2002). Accounting for human polymorphisms predicted to affect protein function. Genome Research.

[B22] Ramensky V, Bork P, Sunyaev S (2002). Human non-synonymous SNPs: server and survey. Nucleic Acids Research.

[B23] PolyPhen and SIFT results. http://pga.gs.washington.edu/data/abo/abobg.pph-sift.txt.

[B24] Metagenomics Prototype Web Tools. http://durian.jgi-psf.org/~eszeto/metag-web/pub/.

[B25] EcoSNP-VISTA program. http://genome.lbl.gov/vista/EcoSNP-VISTA/.

[B26] Huber T, Faulkner G, Hugenholtz P (2004). Bellerophon: A program to detect chimeric sequences in multiple sequence alignments. Bioinformatics.

